# Isolinderalactone suppresses the progression of cholangiocarcinoma by modulating the CARMA1-BCL10-MALT1 signalosome

**DOI:** 10.1016/j.jbc.2026.113162

**Published:** 2026-05-15

**Authors:** Wangyang Chen, Dongchao Xu, Qiang Liu, Xuehui Wang, Chenshan Xu, Yuanling Zhu, Wenjun Lin, Hongchen Zhang, Xiaofeng Zhang, Jianfeng Yang, Hongzhang Shen

**Affiliations:** 1 Department of Gastroenterology, Affiliated Hangzhou First People's Hospital, Westlake University School of Medicine, Hangzhou, Zhejiang Province, China; 2 Hangzhou Institute of Digestive Diseases, Hangzhou, Zhejiang Province, China; 3 Key Laboratory of Integrated Traditional Chinese and Western Medicine for Biliary and Pancreatic Diseases of Zhejiang Province, Hangzhou, Zhejiang Province, China; 4 Key Laboratory of Clinical Cancer Pharmacology and Toxicology Research of Zhejiang Province, Hangzhou, Zhejiang Province, China

**Keywords:** CARMA1-BCL10-MALT1 signalosome, cholangiocarcinoma, isolinderalactone, NF-κB signaling pathway, ubiquitination

## Abstract

Cholangiocarcinoma (CCA) is a highly aggressive malignancy with poor prognosis and limited therapeutic options. While isolinderalactone (ILL) has shown anticancer potential, its effects and underlying mechanisms in CCA remain unclear. This study investigated the anti-CCA activity of ILL and elucidated its molecular mechanisms. ILL significantly inhibited CCA cell viability, proliferation, migration, and invasion, while also inducing G0/G1 cell cycle arrest and promoting apoptosis. Transcriptomic and biochemical analyses revealed that ILL suppressed the NF-κB signaling pathway by inhibiting IκB phosphorylation and p65 activation. Mechanistically, ILL was found to directly bind to BCL10, a key adaptor protein. This interaction disrupted the formation of the CARMA1-BCL10-MALT1 signalosome and consequently reduced BCL10 ubiquitination. Functional experiments confirmed this mechanism: BCL10 knockdown mimicked ILL's inhibitory effects, while a BCL10 mutation abolished them. Furthermore, activation of NF-κB with diprovocim partially reversed ILL's suppressive effects. In a xenograft nude mouse model, ILL effectively reduced tumor growth without significant toxicity and suppressed NF-κB activation in tumor tissues. These findings demonstrate that Isolinderalactone suppresses CCA progression by modulating the CARMA1-BCL10-MALT1 signalosome and BCL10 ubiquitination to regulate the NF-κB pathway, supporting its potential as a promising therapeutic candidate for CCA.

Cholangiocarcinoma (CCA), arising from the biliary epithelium, is recognized for its highly aggressive clinical behavior (1). The incidence of CCA displays a marked geographical disparity, with the highest prevalence concentrated in East Asia, and recent reports indicate a disconcerting upward trend in its global occurrence. CCA is distinguished by its profound invasiveness, propensity for early lymphatic metastasis, and consequently, a poor prognosis, which is reflected in a very low five-year survival rate (2). The clinical management of CCA presents formidable challenges. The absence of distinctive clinical manifestations at early stages frequently postpones diagnosis, leading to detection only after disease advancement, when curative surgery is no longer feasible (3). Moreover, the therapeutic landscape is complicated by significant tumor heterogeneity, encompassing a complex array of genetic and epigenetic alterations that hinder the development of broadly effective targeted treatments (4, 5). These obstacles highlight an urgent necessity to deepen our comprehension of the molecular mechanisms that drive CCA progression and to identify novel therapeutic vulnerabilities and corresponding drug candidates.

Natural products constitute an abundant source for the identification of novel therapeutic agents. Numerous bioactive compounds from botanical sources have shown considerable oncological potential, attributable to their novel chemical scaffolds, multi-targeted modes of action, and often favorable safety profiles (6, 7, 8). Among these is isolinderalactone (ILL), a sesquiterpenoid lactone derived from plants such as *Lindera aggregata* (9). Previous research has established the extensive pharmacological activities of ILL, particularly its potent anti-inflammatory properties (10, 11, 12). More recently, its potential as an anticancer agent has attracted significant interest, with studies demonstrating its capacity to inhibit proliferation, migration, and invasion in diverse cancer types, including colorectal, nonsmall cell lung, pancreatic, and glioblastoma (13, 14, 15, 16). Specifically, ILL effectively inhibits glioblastoma growth by suppressing angiogenesis, downregulating HIF-1α and HIF-2α proteins, and impeding vascular endothelial growth factor expression along with its downstream phosphorylation of vascular endothelial growth factor receptor 2 (13). In colorectal cancer, ILL exerts its tumor-suppressive effects by inducing ROS-mediated apoptosis through the activation of the JNK–p38 MAPK signaling pathways (14). Furthermore, ILL hinders the progression of pancreatic ductal adenocarcinoma by triggering endoplasmic reticulum stress *via* the p38 MAPK pathway, thereby inhibiting epithelial-mesenchymal transition (EMT) and cell proliferation (15). Additionally, in nonsmall cell lung cancer, ILL induces cell cycle arrest by upregulating p21 expression and promotes apoptosis in A549 cells *via* the Fas/sFasL apoptotic system (16). Nevertheless, the therapeutic efficacy of ILL against CCA and its associated molecular mechanisms have yet to be elucidated, representing a promising avenue for investigation.

The NF-κB pathway serves as a central mediator controlling inflammation, immune activity, cell growth, and survival processes (17). NF-κB signaling has been recognized as a hallmark of cancer biology, promoting tumor maintenance through anti-apoptotic signaling, proliferative endurance, angiogenesis, and metastatic dissemination (18). As such, targeting the NF-κB pathway has emerged as a compelling strategy for anticancer drug discovery. Canonical activation of this pathway is contingent upon the assembly of a critical signaling platform known as the CARMA-BCL10-MALT1 (CBM) complex (19, 20). In this tripartite complex, B-cell lymphoma 10 (BCL10) functions as a crucial adaptor protein, bridging the upstream CARMA protein to the downstream effector MALT1. A key regulatory event in this signal transduction is the activating ubiquitination of BCL10. In contrast to the conventional ubiquitin tagging that directs substrates toward degradation, this particular modification generates a molecular platform enabling the assembly and activation of the IKK complex (21). IκBα is first phosphorylated and subsequently degraded, a sequence of events that removes cytoplasmic inhibition of NF-κB and facilitates its nuclear localization, culminating in transcriptional activation. Therefore, interventions that disrupt CBM complex assembly or block BCL10 ubiquitination are regarded as potential strategies to inhibit NF-κB signaling.

Here, we report the initial in-depth characterization of ILL’s pharmacological effects on cholangiocarcinoma progression. We assessed the influence of ILL on a spectrum of malignant phenotypes across multiple CCA cell lines. Employing a combination of transcriptomic profiling and molecular biology validation, we further explored the key signaling cascades and direct molecular targets responsible for ILL's therapeutic effects. ILL treatment was found to significantly inhibit tumor growth in both cultured CCA cells and xenograft models, and mechanistic analyses uncovered a previously unrecognized pathway mediating these effects. Our findings reveal that ILL binds directly to BCL10, destabilizing the CBM complex and suppressing BCL10 ubiquitination, thereby blocking NF-κB activation. Collectively, these results establish ILL as a potential therapeutic candidate for CCA and provide mechanistic insights into its mode of action.

## Results

### ILL inhibits CCA cell proliferation and viability

**Figure 1:**
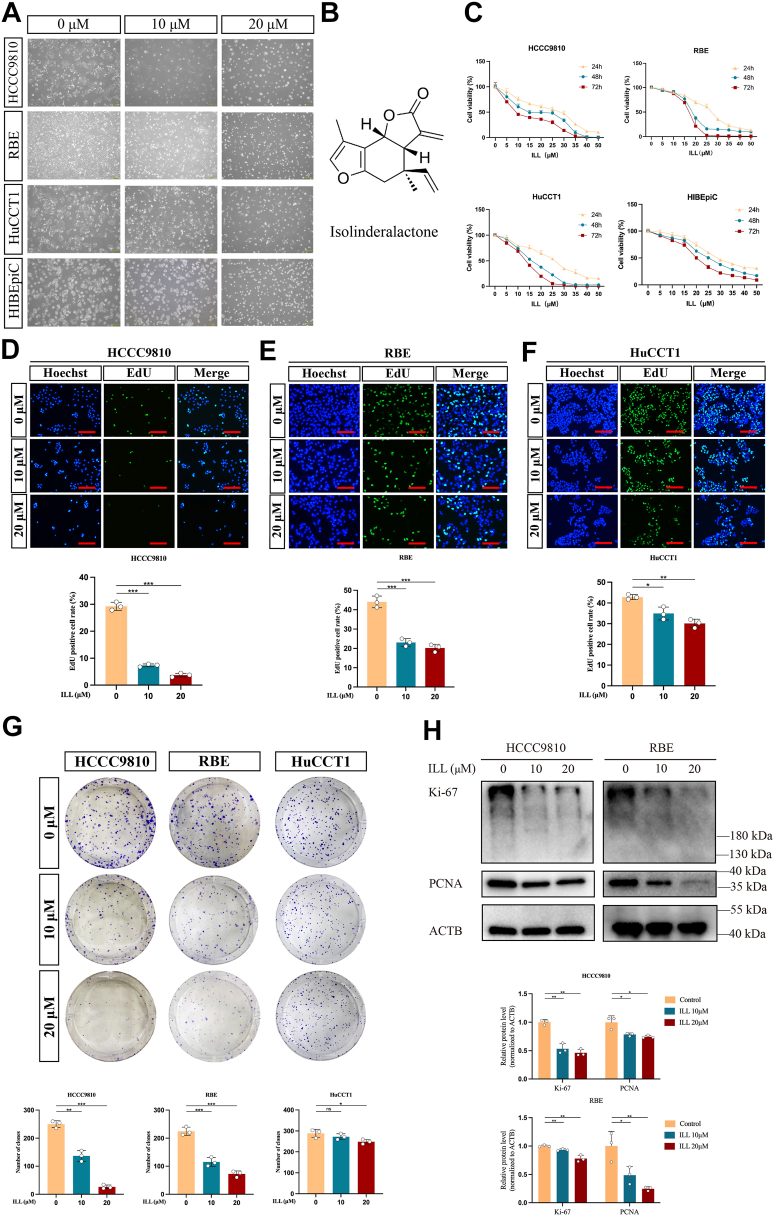
**ILL suppresses proliferation and viability of CCA cells.***A*, morphology of CCA cell lines (HCCC9810, RBE, and HuCCT1) and normal human biliary epithelial cells (HIBEpiC) treated with different concentrations of ILL (0, 10, and 20 μm) for 24 h. Scale bars represent 500 μm. *B*, the chemical structure of ILL. *C*, the viability of HCCC9810, RBE, HuCCT1, and HIBEpiC cells treated with increasing concentrations of ILL for 24, 48, and 72 h was detected by CCK-8 assays. *D* and *F*, the EdU staining detected by fluorescence microscopy demonstrated that the proliferation of CCA cells was inhibited with the treatment of ILL dose-dependently (Scale bars represent 200 μm). And the EdU positive rate of CCA cells (mean ± SD; one-way ANOVA followed by Bonferroni *post hoc* test; n = 3 biologically independent experiments). *G*, the long-term effect of ILL on CCA cells was detected by a colony formation assay. And the number of CCA cell clones (mean ± SD; one-way ANOVA followed by Bonferroni *post hoc* test; n = 3 biologically independent experiments). *H*, the expressions of Ki-67 and PCNA in CCA cells treated with different concentrations of ILL were detected by Western blot and quantified (mean ± SD; one-way ANOVA followed by Bonferroni *post hoc* test; n = 3 biologically independent experiments). ∗*p* < 0.05, ∗∗*p* < 0.01, ∗∗∗*p* < 0.001.

To assess the anticancer potential of ILL, its impact on CCA cell viability was investigated. Microscopic examination revealed a dose-dependent reduction in cell density and discernible alterations in cellular morphology in CCA cells and normal human biliary epithelial cells (HIBEpiC) upon ILL treatment (Fig. 1, *A* and *B*). Notably, HIBEpiC maintained their typical morphology even at concentrations that markedly affected CCA cells, indicating a potential selective effect of ILL. CCK-8 assays were employed to assess the effects of ILL on both CCA cells and HIBEpiC. Results showed a concentration- and time-dependent decrease in viability across all three CCA cell lines, whereas HIBEpiC were also affected by ILL but exhibited relatively lower sensitivity under the same treatment conditions (Fig. 1*C*). We next investigated the antiproliferative mechanism of ILL using EdU incorporation and colony formation assays. As DNA synthesis during the S phase is a hallmark of cellular proliferation, EdU staining was employed. Quantification of EdU-positive cells showed that ILL treatment led to a concentration-dependent decrease in DNA replication, highlighting its capacity to impede cell cycle progression in CCA cells (Fig. 1, *D*–*F*). Concurrently, ILL treatment markedly impaired the clonogenic survival and long-term proliferative capacity of all three CCA cell lines, as evidenced by a reduction in colony numbers (Fig. 1*G*). Furthermore, Western blot analysis corroborated these phenotypic observations, showing that ILL treatment induced a significant downregulation of the proliferation markers Ki-67 and PCNA (Fig. 1*H*).

### ILL suppresses the migratory and invasive capabilities of CCA cells

**Figure 2:**
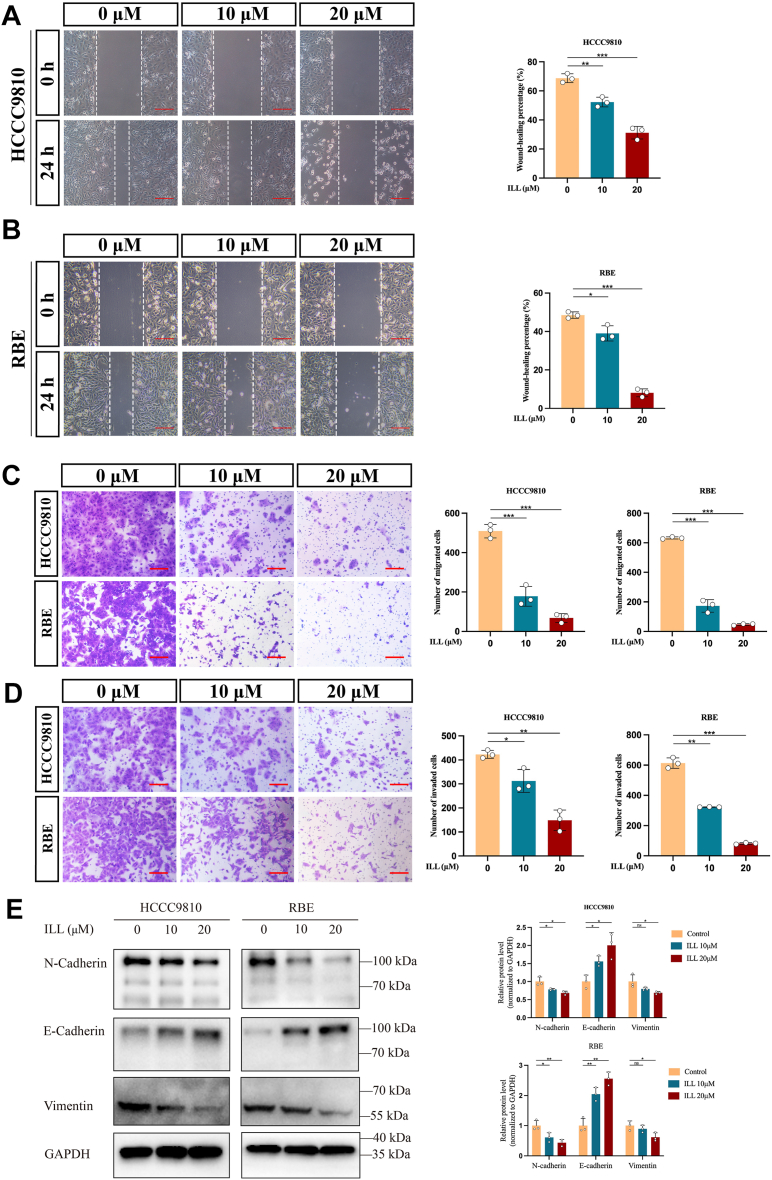
**ILL suppresses migration and invasion of CCA cells.***A* and *B*, the wound-healing assays demonstrated that the migration of CCA cells inhibited the treatment of ILL dose-dependently (mean ± SD; one-way ANOVA followed by Bonferroni *post hoc* test; n = 3 biologically independent experiments). *C*, in the transwell migration assays, the migrated CCA cells were reduced with the treatment of different concentration of ILL (mean ± SD; one-way ANOVA followed by Bonferroni *post hoc* test; n = 3 biologically independent experiments). *D*, in the transwell invasion assays, the invaded CCA cells were reduced with the treatment of different concentration of ILL (mean ± SD; one-way ANOVA followed by Bonferroni *post hoc* test; n = 3 biologically independent experiments). *E*, the expressions of N-cadherin, E-cadherin, and vimentin in CCA cells treated with different concentrations of ILL were detected by Western blot and quantified (mean ± SD; one-way ANOVA followed by Bonferroni *post hoc* test; n = 3 biologically independent experiments). ∗*p* < 0.05, ∗∗*p* < 0.01, ∗∗∗*p* < 0.001.

Given that tumor metastasis is a key determinant of cancer progression, we assessed the impact of ILL on the migratory and invasive capabilities of CCA cells. A two-dimensional wound-healing assay demonstrated that ILL treatment dose-dependently impeded the migratory capacity of CCA cells, with the 20 μm concentration causing the most substantial inhibition of wound closure (Fig. 2, *A* and *B*). The inhibitory effect of ILL on cellular motility was further validated *via* three-dimensional transwell assays, which demonstrated a progressive reduction in migrating cells in response to escalating ILL concentrations (0, 10, and 20 μm) (Fig. 2*C*). Transwell invasion assays employing Matrigel-coated inserts demonstrated that increasing doses of ILL effectively curtailed the invasive potential of HCCC9810 and RBE cells (Fig. 2*D*). Given that the EMT is a crucial mechanism driving cancer cell migration and invasion, we next evaluated the expression of canonical EMT markers to explore the underlying molecular basis. Western blot analysis further revealed that ILL dose-dependently decreased the expression of the mesenchymal markers N-Cadherin and Vimentin, along with increased levels of the epithelial marker E-Cadherin (Fig. 2*E*).

### ILL inhibited CCA cell progression by promoting cell cycle arrest and apoptosis

**Figure 3:**
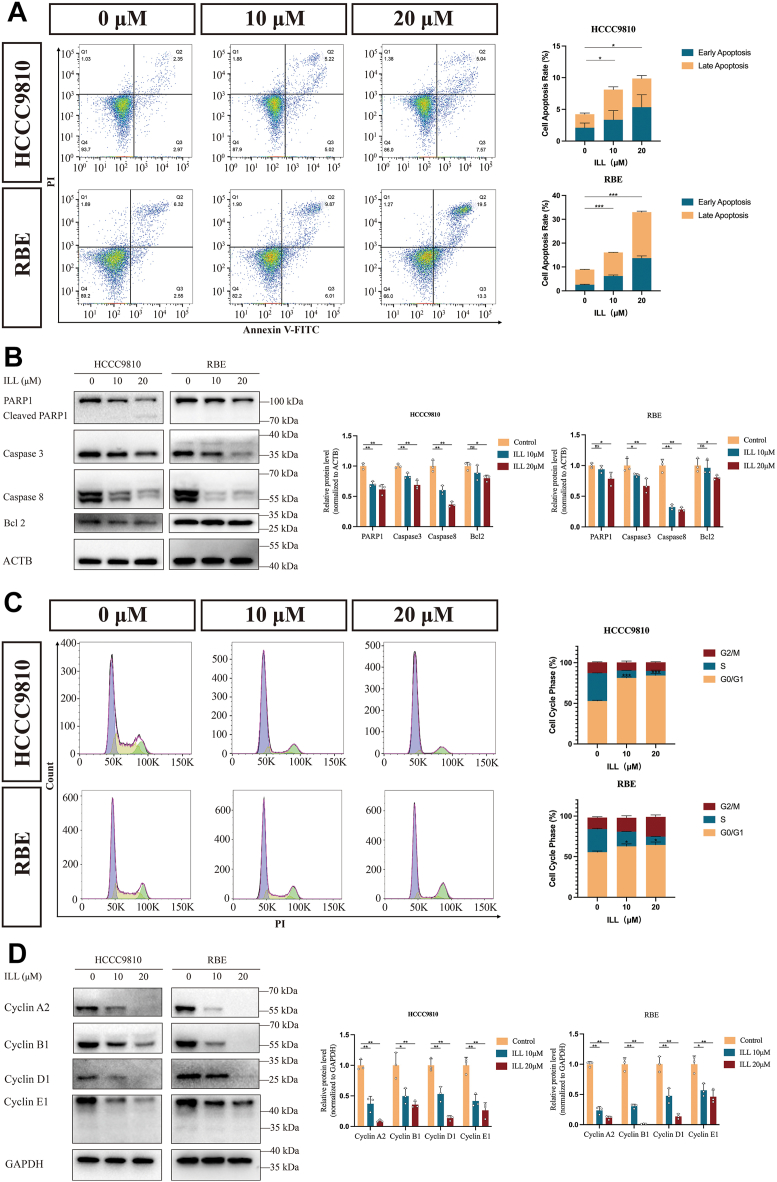
**ILL induced apoptosis and induced CCA cells cycle arrest in G0/G1.***A*, flow cytometry analysis of CCA cell apoptosis was performed by Annexin V-PI double staining (mean ± SD; one-way ANOVA followed by Bonferroni *post hoc* test; n = 3 biologically independent experiments). *B*, the expressions of cell cycle–related proteins in CCA cells treated with different concentrations of ILL were detected by Western blot and quantified (mean ± SD; one-way ANOVA followed by Bonferroni *post hoc* test; n = 3 biologically independent experiments). *C*, flow cytometric analysis of CCA cell cycle with the treatment of ILL (mean ± SD; one-way ANOVA followed by Bonferroni *post hoc* test; n = 3 biologically independent experiments). *D*, the expressions of cell apoptosis–related proteins in CCA cells treated with different concentrations of ILL were detected by Western blot and quantified (mean ± SD; one-way ANOVA followed by Bonferroni *post hoc* test; n = 3 biologically independent experiments). ∗*p* < 0.05, ∗∗*p* < 0.01, ∗∗∗*p* < 0.001.

To elucidate the mechanisms underlying ILL's antiproliferative effects, we investigated its impact on cell cycle progression and apoptosis. Annexin V-FITC and propidium iodide (PI) double staining followed by flow cytometry revealed that treatment with 10 μm or 20 μm ILL markedly increased the proportion of apoptotic cells at both early and late stages compared to controls (Fig. 3*A*). To substantiate the induction of apoptosis, Western blot assays were performed, demonstrating that ILL promoted cleavage of PARP1, Caspase-3, and Caspase-8, accompanied by downregulation of Bcl-2 (Fig. 3*B*). Further cell cycle assessment *via* flow cytometry indicated that ILL significantly arrested CCA cells in G0/G1, with a concomitant reduction in S-phase cell populations (Fig. 3*C*). Western blot analysis further validated the cell cycle blockade, demonstrating modulation of key regulatory proteins indicative of G0/G1 arrest (Fig. 3*D*). These findings suggest that ILL suppresses CCA cell proliferation by concurrently inducing apoptosis and G0/G1 phase cell cycle arrest.

### ILL suppresses cholangiocarcinoma cell proliferation by inhibiting the NF-κB signaling pathway

**Figure 4:**
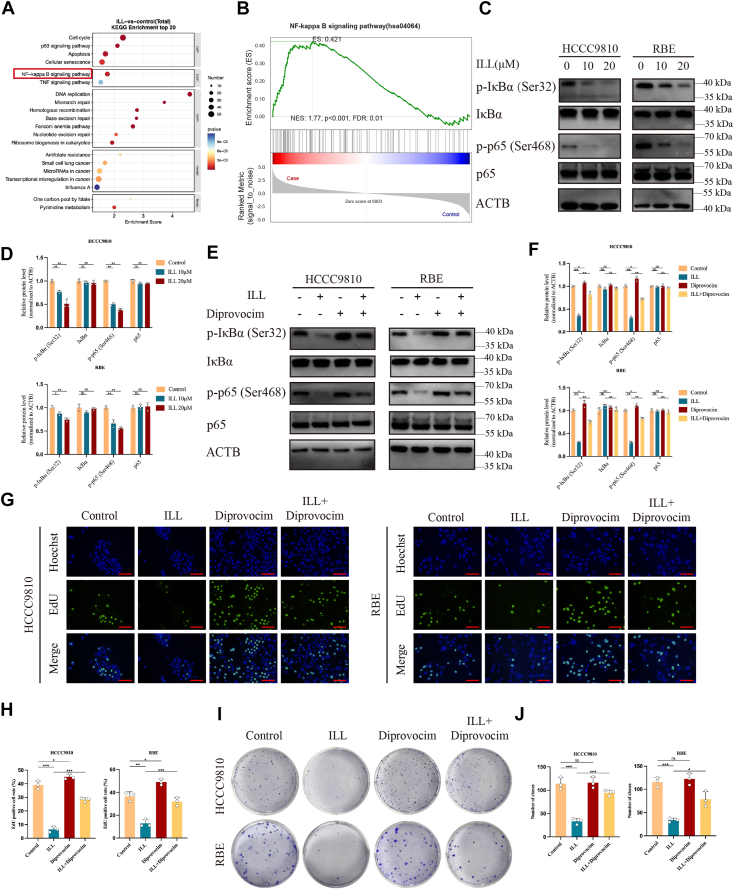
**ILL suppresses the NF-κB signaling pathway in CCA cells.***A*, KEGG pathway enrichment analysis of differentially expressed genes between control and ILL-treated groups. *B*, GSEA analysis showing enrichment of the NF-κB signaling pathway. *C* and *D*, the expressions of p-IκBα (Ser32), IκBα, p-p65 -(Ser468), and p65 in CCA cells treated with different concentrations of ILL were detected by Western blot and quantified (mean ± SD; one-way ANOVA followed by Bonferroni *post hoc* test; n = 3 biologically independent experiments). *E* and *F*, Western blot analysis and quantification of p-IκBα (Ser32), IκBα, p-p65 (Ser468), and p65 in CCA cells treated with ILL, diprovocim, or the combination of ILL and diprovocim (mean ± SD; one-way ANOVA followed by Bonferroni *post hoc* test; n = 3 biologically independent experiments). *G* and *H*, EdU staining and positive rate analysis in CCA cells treated with ILL, diprovocim, or the combination of ILL and diprovocim (mean ± SD; one-way ANOVA followed by Bonferroni *post hoc* test; n = 3 biologically independent experiments). Scale bars represent 200 μm. *I* and *J*, colony formation assays and quantification of colony numbers in CCA cells (mean ± SD; one-way ANOVA followed by Bonferroni *post hoc* test; n = 3 biologically independent experiments). ∗*p* < 0.05, ∗∗*p* < 0.01, ∗∗∗*p* < 0.001.

To explore the molecular mechanisms by which ILL exerts its effects, transcriptomic profiling was conducted in CCA cells following ILL treatment. Kyoto Encyclopedia of Genes and Genomes (KEGG) pathway analysis indicated that the differentially expressed genes (DEGs) were predominantly associated with multiple cancer-related pathways, with NF-κB signaling among the most significantly impacted (Fig. 4*A*). Gene set enrichment analysis (GSEA) further confirmed a significant positive enrichment of this pathway in the ILL-treated group (NES = 1.77, *p* < 0.001, FDR = 0.01), suggesting that ILL may modulate CCA cell behavior by regulating NF-κB signaling (Fig. 4*B*). To validate the changes in NF-κB signaling, we examined both the total and phosphorylated levels of IκBα and p65 at specific regulatory sites. Specifically, we evaluated IκBα phosphorylation at Serine 32 (Ser32), a modification that canonically triggers its proteasomal degradation and the subsequent nuclear translocation of NF-κB dimers. Concurrently, we assessed p65 phosphorylation at Serine 468 (Ser468), an activating event that enhances its transcriptional capacity. Western blot results indicated that escalating ILL concentrations resulted in a marked downregulation of p-IκBα (Ser32) and p-p65 (Ser468), without altering the total abundance of these proteins (Fig. 4, *C* and *D*). These findings indicate that ILL suppresses NF-κB activation by preventing IκBα degradation and reducing p65 transcriptional activation. To further assess whether the inhibitory effects of ILL could be reversed by an NF-κB agonist, cells were treated with diprovocim. Diprovocim, a synthetic TLR1/2 agonist, activates NF-κB signaling through the MyD88-dependent pathway, leading to IκBα phosphorylation and subsequent p65 activation. Diprovocim administration markedly enhanced the phosphorylation of IκBα and p65. Cotreatment with ILL and diprovocim effectively restored phosphorylation levels, approaching those observed in the control group, suggesting that diprovocim can counteract the inhibitory effects of ILL on NF-κB signaling (Fig. 4, *E* and *F*). Subsequently, we evaluated whether these molecular alterations translated into phenotypic changes by performing EdU incorporation and colony formation assays. EdU incorporation assays demonstrated that ILL markedly decreased the proportion of EdU-positive cells in both CCA cell lines, reflecting suppressed DNA synthesis, while diprovocim treatment significantly enhanced EdU incorporation. Cotreatment with diprovocim restored the EdU-positive rates that were suppressed by ILL (Fig. 4, *G* and *H*). Similarly, colony formation assays showed that ILL significantly impaired clonogenic capacity, diprovocim enhanced colony formation, and cotreatment with ILL and diprovocim effectively reversed the inhibitory effects of ILL on clonogenicity (Fig. 4, *I* and *J*). Given the inherent pleiotropic effects of TLR1/2 activation, we sought to corroborate these functional rescues using an additional distinct NF-κB activator, PapRIV. Consistent with the diprovocim data, cotreatment with PapRIV effectively reversed the ILL-induced inhibition of EdU incorporation and colony formation, thereby further substantiating the functional reliance on the NF-κB axis (Fig. S1). To further assess the transcriptional effects of NF-κB inhibition, we measured the mRNA levels of NF-κB target genes by quantitative PCR. ILL treatment significantly reduced their expression in both HCCC9810 and RBE cells, indicating suppression of NF-κB–dependent transcriptional activity (Fig. S2).

### ILL suppresses CCA cell proliferation through binding to BCL10

**Figure 5:**
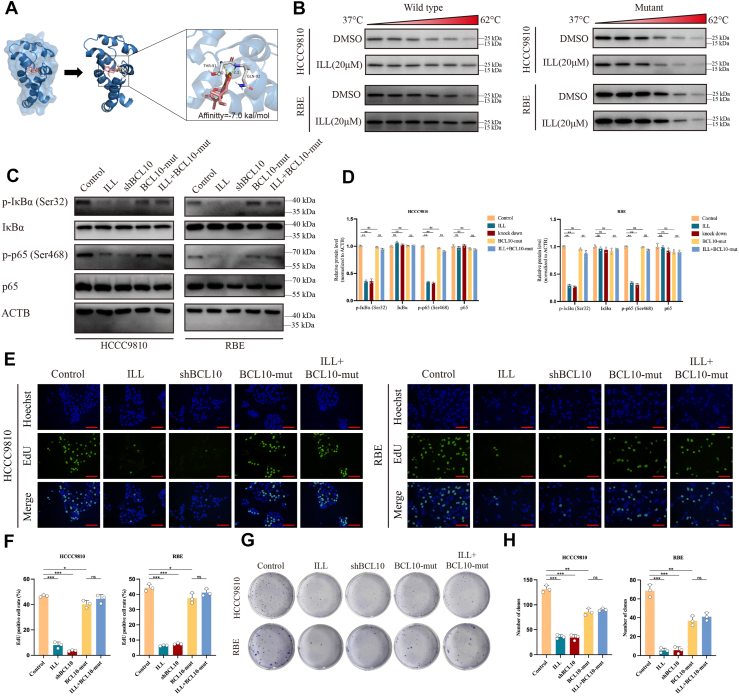
**ILL suppresses CCA cell proliferation through binding to BCL10.***A*, molecular docking analysis showing the predicted binding mode of ILL with BCL10 and the binding energy is −7.0 kcal/mol. *B*, CETSA of WT BCL10 and mutant BCL10 in CCA cells treated with DMSO or ILL (20 μm) across a temperature gradient from 37 to 62 °C. *C* and *D*, Western blot and quantification of p-IκBα (Ser32), IκBα, p-p65 (Ser468), and p65 in CCA cells under the indicated treatments: control, ILL (20 μm), shBCL10, BCL10-mut, and ILL plus BCL10-mut (mean ± SD; one-way ANOVA followed by Bonferroni *post hoc* test; n = 3 biologically independent experiments). *E* and *F*, EdU staining and positive rate analysis of CCA cells under the indicated treatments (mean ± SD; one-way ANOVA followed by Bonferroni *post hoc* test; n = 3 biologically independent experiments). Scale bars represent 200 μm. *G* and *H*, colony formation assays and quantification of colony numbers in CCA cells under the indicated treatments (mean ± SD; one-way ANOVA followed by Bonferroni *post hoc* test; n = 3 biologically independent experiments). ∗*p* < 0.05, ∗∗*p* < 0.01, ∗∗∗*p* < 0.001.

Given that the CBM signalosome represents a central upstream regulatory hub of canonical NF-κB activation in cancer cells, we focused our target screening on key components of this axis, together with the downstream IKK complex. To identify the potential direct molecular target of ILL, we first conducted molecular docking analysis targeting key components of the CBM signalosome and the downstream IKK complex. The results revealed that while ILL exhibited potential interactions with several proteins, its calculated binding affinity for BCL10 was consistently the highest (Figs. 5*A* and S3). ILL can stably bind to BCL10 at Thr-91 and Gln-92, forming hydrogen bonds with lengths of 2.3 Å and 2.2 Å, respectively, and exhibiting a binding energy of −7.0 kcal/mol. To validate the functional relevance of these predicted binding sites, we generated a BCL10 mutant (BCL10-mut) harboring point mutations at the key residues Thr-91 and Gln-92 identified by molecular docking. To confirm the cellular significance of this interaction, a cellular thermal shift assay (CETSA) was conducted to evaluate the thermal stability of MALT1, CARMA1, IKKα, IKKβ, IKKγ, as well as WT and mutant BCL10 at putative binding sites (Figs. 5*B* and S3). Notably, ILL treatment failed to induce significant or consistent thermal stabilization in MALT1, CARMA1, or the IKK complex subunits. In contrast, ILL significantly increased the thermal resilience of WT BCL10 over a temperature gradient in both HCCC9810 and RBE cells when compared to vehicle controls. Furthermore, ILL treatment did not alter the thermal stability of the BCL10 mutant at putative binding sites (Fig. 5*B*). These findings suggest that ILL directly and specifically binds to and stabilizes BCL10. To further investigate the role of BCL10 in ILL-mediated inhibition of the NF-κB pathway, stable BCL10 knockdown (shBCL10) cell lines were first established using shRNA. Subsequently, shBCL10 cells were transfected with plasmids encoding BCL10-mut, generating a rescue model for functional analysis. Western blot analysis revealed that shBCL10 significantly reduced p-IκBα (Ser32) and p-p65 (Ser468) levels without affecting the total levels of IκBα or p65, mirroring the effects observed with ILL treatment. In contrast, no significant changes in p-IκBα (Ser32) and p-p65 (Ser468) were detected in the mutant or ILL + BCL10-mut groups compared with the control group (Fig. 5, *C* and *D*). Notably, the BCL10-mut failed to restore NF-κB signaling activity in shBCL10 cells, indicating that mutations at Thr-91 and Gln-92 impair BCL10-mediated NF-κB signaling, despite not affecting its basal interaction with CBM complex components. Phenotypic analysis *via* EdU incorporation indicated that ILL treatment effectively lowered the percentage of EdU-positive cells, mirroring the suppressive effect observed with shBCL10 (Fig. 5, *E* and *F*). However, the BCL10-mut and ILL + BCL10-mut groups showed no notable difference from the control, indicating that the loss of BCL10 function negates ILL’s inhibitory effect on DNA synthesis. Consistently, colony formation assays revealed that both ILL and shBCL10 significantly reduced colony numbers, whereas the mutant and ILL + BCL10-mut groups displayed colony formation similar to the control (Fig. 5, *G* and *H*).

### ILL inhibits NF-κB signaling by disrupting BCL10 complex formation and inhibiting its ubiquitination

**Figure 6:**
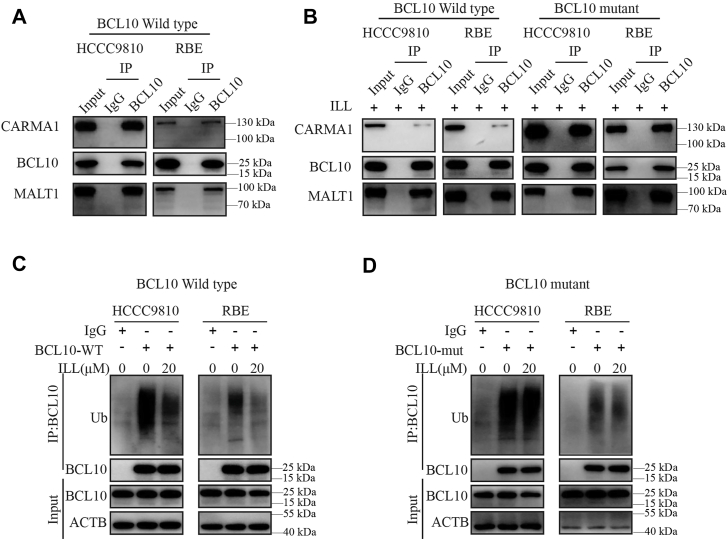
**ILL inhibits NF-κB signaling by modulating BCL10 complex formation and inhibiting its ubiquitination.***A*, co-IP analysis of interactions between BCL10 and CARMA1 or MALT1 in CCA cells expressing WT BCL10. *B*, co-IP analysis of interactions between BCL10 and CARMA1 or MALT1 in CCA cells expressing WT BCL10 or mutant BCL10 with the effect of ILL. *C* and *D*, ubiquitination of BCL10 in BCL10-WT–expressing or BCL10-mut–expressing CCA cells treated with 0 or 20 μm ILL for 24 h.

NF-κB signaling is tightly regulated by the CARMA1-BCL10-MALT1 signalosome, which serves as a scaffold to recruit downstream signaling components, and the ubiquitination of BCL10 plays a critical role in modulating CBM complex stability and NF-κB activation (21, 22, 23). To further clarify how ILL exerts its effects, we assessed its influence on the formation of the CBM complex and the ubiquitination status of BCL10. First, co-immunoprecipitation (co-IP) assays confirmed that BCL10-WT readily interacts with both CARMA1 and MALT1, establishing the formation of the CBM complex under basal conditions in HCCC9810 and RBE cells (Fig. 6*A*). However, upon treatment with ILL, BCL10-WT showed a significant reduction in its interaction specifically with CARMA1, while the association with MALT1 remained largely unaffected. In contrast, in the BCL10-mut cells, ILL treatment did not disrupt CBM complex formation, suggesting that the interaction between BCL10 and CARMA1 is crucial for ILL's inhibitory effect on complex assembly (Fig. 6*B*). To exclude the possibility that the BCL10 mutation intrinsically disrupts CBM complex formation, we performed additional co-IP assays in the absence of ILL treatment. The results showed that BCL10-mut retained its ability to interact with both CARMA1 and MALT1 in HCCC9810 and RBE cells, comparable to WT BCL10 (Fig. S4). These findings indicate that the mutations at Thr-91 and Gln-92 do not impair the basal assembly of the CBM complex. Therefore, the lack of response to ILL in BCL10-Mut cells is attributable to the loss of ILL binding rather than an inherent defect in complex formation. Next, we examined the effect of ILL on BCL10 ubiquitination. In BCL10-WT cells, treatment with 20 μm ILL significantly decreased the ubiquitination of BCL10 (Fig. 6*C*). In contrast, in BCL10-mut cells, the levels of BCL10 ubiquitination remained unchanged upon ILL treatment (Fig. 6*D*). To further distinguish between different types of ubiquitination, we performed ubiquitination assays using ubiquitin mutants that selectively permit K48- or K63-linked chain formation. The results showed that ILL markedly reduced K63-linked ubiquitination of BCL10, while having minimal effect on K48-linked ubiquitination (Fig. S5). These findings indicate that ILL preferentially inhibits the activating ubiquitination of BCL10 rather than affecting its proteasomal degradation. These findings collectively indicate that ILL suppresses NF-κB signaling by disrupting the BCL10–CARMA1 interaction, thus compromising the integrity of the CBM complex and inhibiting BCL10 ubiquitination.

### ILL suppresses tumor growth and NF-κB signaling *in vivo*

**Figure 7:**
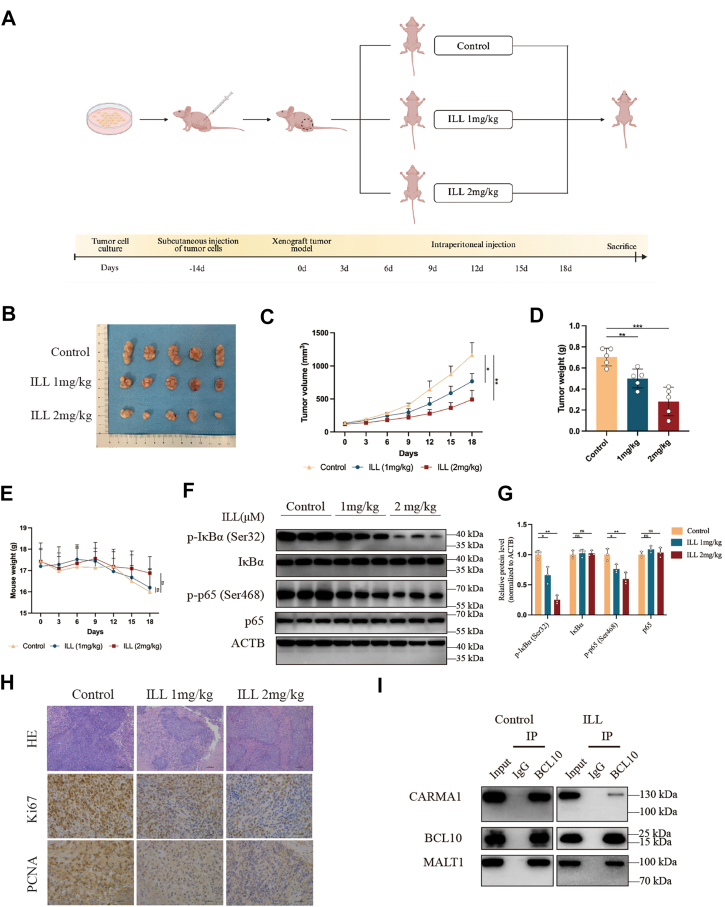
**ILL suppresses tumor growth and NF-κB signaling *in vivo*.***A*, schematic diagram of the experimental design for the xenograft mouse model. *B*, images of excised tumors from each group at the end of the experiment. *C*, the progression of tumor volume in each group (mean ± SD; one-way ANOVA followed by Bonferroni *post hoc* test; n = 5 biologically independent experiments). *D*, the tumor weights at the end of the experiment (mean ± SD; one-way ANOVA followed by Bonferroni *post hoc* test; n = 5 biologically independent experiments). *E*, body weight changes of mice during the period (mean ± SD; one-way ANOVA followed by Bonferroni *post hoc* test; n = 5 biologically independent experiments). *F* and *G*, the expressions of p-IκB, IκB, p-p65, and p65 in tumor tissues from each group were detected by Western blot and quantified (mean ± SD; one-way ANOVA followed by Bonferroni *post hoc* test; n = 3 biologically independent experiments). *H*, histological analysis of tumor tissues by H&E staining (Scale bars represent 50.8 μm), and IHC staining for Ki-67 and PCNA (Scale bars represent 12.5 μm). *I*, co-immunoprecipitation analysis of BCL10 interaction with CARMA1 and MALT1 in tumor lysates. ∗*p* < 0.05, ∗∗*p* < 0.01, ∗∗∗*p* < 0.001.

To evaluate the *in vivo* activity of ILL, a subcutaneous xenograft model was established in nude mice, followed by administration of ILL at doses of 1 and 2 mg/kg (Fig. 7, *A* and *B*). The selected doses of ILL (1 and 2 mg/kg) were based on previously reported *in vivo* studies of ILL with antitumor activity and were further supported by our preliminary tolerability observations, as evidenced by stable body weight throughout the treatment period (13, 15). Tumor length and width were measured using a digital caliper, with length defined as the longest diameter and width as the perpendicular shorter diameter, and tumor volumes were calculated using the formula (length × width^2^)/2. Tumor volumes were measured at regular intervals, revealing significant inhibition of tumor growth in both ILL-treated groups, with more pronounced effects observed at the higher dose (Fig. 7*C*). At sacrifice, tumor weights were also significantly reduced in ILL-treated mice compared to controls (Fig. 7*D*). No significant changes in body weight were observed across the groups, indicating that ILL treatment did not induce substantial systemic toxicity (Fig. 7*E*). Histological analysis of tumor tissues, performed using H&E staining and immunohistochemistry for Ki67 and PCNA, revealed decreased Ki67 and PCNA expression in ILL-treated mice, suggesting inhibition of tumor cell proliferation (Fig. 7*F*). Moreover, Western blotting revealed a substantial decrease in p-IκBα (Ser32) and p-p65 (Ser468) protein levels in tumor tissues from mice treated with ILL (Fig. 7, *G* and *H*). Co-IP analysis of tumor lysates further showed that ILL treatment reduced the interaction of BCL10 with CARMA1 and MALT1, indicating disruption of CBM complex assembly *in vivo* (Fig. 7*I*).

## Discussion

CCA is a highly aggressive malignancy of the gastrointestinal tract, characterized by complex pathogenesis and poor clinical outcomes that have remained largely unchanged for decades. This stagnation is attributable to both the intricate molecular mechanisms underlying the disease and the scarcity of effective therapeutic options. Therefore, the development of innovative strategies targeting critical oncogenic pathways is urgently needed to overcome this therapeutic impasse. This study systematically investigates the potential effect of the natural product ILL as a novel anti-CCA drug candidate and, for the first time, comprehensively elucidates its molecular mechanism of action. Our results indicate that ILL exhibits strong antitumor effects by directly interacting with BCL10, leading to suppression of canonical NF-κB pathway activation. This mechanism was confirmed using both *in vitro* experiments and *in vivo* preclinical models, highlighting BCL10 as a previously unrecognized molecular target and positioning ILL as a potential candidate for targeted therapy in cholangiocarcinoma.

Our findings reveal that ILL exerts comprehensive antitumor effects in CCA, not only suppressing cell proliferation and promoting apoptosis but also counteracting EMT, a pivotal process driving invasion and metastasis (24, 25, 26). EMT is a principal driver of poor prognosis in CCA patients (27). Mechanistically, ILL upregulates the epithelial marker E-cadherin while downregulating the mesenchymal markers N-cadherin and vimentin, thereby markedly impairing cellular migratory and invasive capacities. These findings suggest that ILL may not only suppress primary tumor growth but also mitigate metastasis, addressing a central challenge in CCA management.

At the mechanistic level, our study highlights that the anticancer effects of ILL are closely associated with the suppression of NF-κB signaling, a pathway whose constitutive activation is implicated as a critical driver in inflammation-related cancers, including CCA (28, 29). Transcriptomic profiling initially implicated NF-κB suppression in ILL’s mode of action, and functional validation confirmed that NF-κB inhibition is essential for ILL’s antitumor efficacy. This finding underscores that ILL’s effects are not attributable to nonspecific cytotoxicity but are instead due to precise targeting of a validated oncogenic pathway.

One of the key contributions of this work is the identification of BCL10 as a primary intracellular binding partner of ILL. Our approach progressed from *in silico* molecular docking predictions to rigorous experimental validation. The CETSA verified direct drug-target engagement under physiological conditions, demonstrating that ILL binds to WT BCL10 and enhances its thermal stability (30, 31). Genetic validation further confirmed the indispensability of BCL10 for ILL-mediated therapeutic effects. Functioning as a central adaptor in the CBM signalosome, BCL10 has historically been regarded as a challenging target for small-molecule therapeutics owing to the lack of a conventional ligand-binding site (20, 32). Our results position ILL as a small-molecule inhibitor capable of directly engaging BCL10, thereby opening new avenues for drug discovery targeting the CBM complex. Downstream mechanistic studies revealed that ILL–BCL10 binding likely induces conformational changes that disrupt BCL10’s interaction with CARMA1, thereby impeding CBM complex assembly. Co-IP and ubiquitination assays demonstrated that this disruption prevents the activating ubiquitination of BCL10, which is necessary for the recruitment and activation of the IKK complex. This blockade effectively terminates NF-κB signaling at its initiation point.

Despite these promising results, several limitations must be considered. First, although our pharmacological rescue with diprovocim and PapRIV supports NF-κB dependency, these agonists exhibit inherent pleiotropy. While using two distinct activators mitigates this concern, definitively isolating the NF-κB–specific contribution necessitates future genetic interventions. Second, the subcutaneous xenograft model used in this study does not fully replicate the desmoplastic and heterogeneous tumor microenvironment characteristic of human CCA. Future studies should employ more clinically relevant models, such as patient-derived xenografts or orthotopic implantation models, to better mimic the disease’s biology. Third, while animal studies indicate good tolerability, comprehensive pharmacokinetic profiling and toxicological evaluations are necessary before advancing to clinical trials. Finally, although computational docking and mutational analysis suggest a plausible binding site, definitive confirmation of ILL’s interaction with BCL10 will require high-resolution structural studies, such as co-crystallography or advanced site-directed mutagenesis.

In conclusion, this study provides the first comprehensive demonstration of ILL’s mechanism of action as an anti-CCA agent. By directly targeting BCL10, disrupting CBM complex assembly, inhibiting activating ubiquitination, and thereby suppressing NF-κB signaling, ILL inhibits both tumor growth and metastatic potential. This work not only validates BCL10 as a druggable target but also positions ILL as a highly promising natural product for therapeutic development. Future research should focus on optimizing ILL’s pharmacological properties through medicinal chemistry, evaluating combination strategies with existing chemotherapies, and conducting thorough preclinical safety studies to expedite clinical translation.

## Experimental procedures

### Reagent

The compound ILL was obtained from Herbpurify (purity ≥ 98%). The compound was dissolved in dimethyl sulfoxide to create a concentrated stock, which was maintained at 4 °C.

### Cell culture

HCCC9810, RBE, and HuCCT1 CCA cell lines were acquired from the ATCC and the Cell Bank of the Chinese Academy of Sciences. HuCCT1 cells were sustained in Dulbecco’s modified Eagle’s medium, while HCCC9810 and RBE cell lines were cultured in RPMI-1640, both media supplied by Gibco. For maintenance of all cell lines, media were supplemented with 10% fetal bovine serum and 1% penicillin–streptomycin (Solarbio). Cultures were incubated at 37 °C in a humidified chamber with 5% CO_2_.

### Cell viability assay

Cellular responses to ILL treatment were quantified using the CCK-8 kit (Bimake). In 96-well plate format, CCA cells were seeded at 3 × 10^3^ cells per well and treated with escalating concentrations of ILL (0, 5, 10, 15, 20, 25, 30, 35, 40, 50 μm). Following ILL treatment for 24, 48, or 72 h, 10% CCK-8 reagent was added to the wells and incubated at 37 °C for 2 h. The resulting absorbance at 450 nm was measured using a microplate spectrophotometer.

### EdU assay

Cellular growth activity was examined using an EdU Cell Proliferation Kit with Alexa Fluor 488 (Beyotime Biotechnology) according to the supplier’s instructions. CCA cells were plated into 12-well plates (3000 cells per well) and treated with different concentration of ILL for 24 h. EdU was then introduced to label newly synthesized DNA, and nuclei were counterstained with Hoechst 33342.

### Colony formation assay

The ability of CCA cells to form colonies was conducted to quantify clonogenicity. HCCC9810 and RBE cells were seeded at 750 cells per well in 6-well plates and cultured for 72 h to achieve firm attachment. Cells were treated with graded concentrations of ILL for 24 h, after which the medium was replaced with fresh medium. The cultures were maintained for 14 days to allow colony development, followed by fixation with 4% paraformaldehyde and staining with 1% crystal violet (Beyotime Biotechnology).

### Wound-healing assay

Migration of CCA cells was evaluated through a wound-healing assay. CCA cells were seeded at a density of 1 × 10^6^ per well in 6-well plates and grown to form a confluent monolayer. The monolayer underwent 24 h of serum starvation before a linear scratch was introduced using a sterile 200 μl pipette tip. After removal of floating cells, cultures were maintained in serum-free medium containing various ILL concentrations. Images were collected at 0 h and 24 h later, and the migration distance was analyzed with ImageJ (NIH).

### Transwell migration and invasion assays

The extent of cell migration and invasion was measured using transwell chamber systems. For the invasion assessment, the apical side of the upper chambers was coated with Matrigel (Corning). A total of 2 × 10^6^ cells were resuspended in 200 μl of serum-free medium containing ILL (0, 10, or 20 μm) and seeded into the upper chamber, while the lower chamber was filled with medium supplemented with 10% fetal bovine serum to serve as a chemoattractant for migration and invasion. Following 24 h incubation, cells that remained on the upper surface were removed, whereas those that traversed the membrane were fixed with 4% paraformaldehyde, stained with crystal violet, and quantified using ImageJ.

### Flow cytometric analysis of cell cycle and cell apoptosis

For analysis of the cell cycle, CCA cells were seeded in 6-well plates and exposed to different concentrations of ILL for 24 h. Approximately 1 × 10^6^ cells per sample were collected, rinsed with phosphate-buffered saline (PBS), and fixed overnight at 4 °C in 70% ethanol. Fixed cells were then stained with PI from the Cell Cycle Analysis Kit (Beyotime Biotechnology) for 1 h in the dark prior to flow cytometric assessment. For apoptosis detection, cells were plated at 9 × 10^4^ per well, treated under the same conditions, harvested using EDTA-free trypsin, washed, and labeled with Annexin V-FITC and PI according to the kit instructions. Data for both assays were acquired on a BD FACSCanto II cytometer and analyzed with FlowJo software (v10.8.1, BD Biosciences).

### RNA-seq and bioinformatics analysis

RNA-seq was employed to characterize gene expression changes in HCCC9810 cells treated with 20 μm ILL or vehicle for 24 h. Total RNA was extracted using TRIzol (Invitrogen) according to manufacturer guidelines and assessed for integrity with an Agilent 2100 Bioanalyzer. Sequencing libraries were constructed, and data generation was performed by Shanghai Ouyi Biomedical Technology Co, Ltd. DEGs were identified *via* DESeq2 using a q-value cutoff of 0.05 and fold change thresholds of >2 or <0.5. Functional annotation and pathway enrichment analyses were conducted using KEGG and GSEA, with KEGG pathway diagrams created in R and GSEA applied to evaluate enrichment trends among the ranked DEGs.

### Quantitative real-time PCR

Total RNA was reverse-transcribed to complementary DNA using the PrimeScript RT Reagent Kit (Takara). Quantitative real-time PCR was performed using SYBR Green Master Mix (Takara) on a QuantStudio 5 system (Applied Biosystems). ACTB was used as an internal reference, and the sequences of primers for BCL2, GADD45B, TRAF1, and ACTB are provided in Table S2.

### Western blot analysis

To assess protein expression, total lysates from CCA cell cultures or tumor tissues were extracted with RIPA buffer (Beyotime Biotechnology) containing protease and phosphatase inhibitors (Thermo Fisher Scientific). Protein concentrations were measured using a bicinchoninic acid assay. Equal protein amounts were loaded onto SDS-PAGE gels, separated, and transferred onto polyvinylidene difluoride membranes. After a 1 h blocking step with 5% nonfat milk in TBST (0.1% Tween-20), membranes were incubated overnight at 4 °C with primary antibodies, followed by a 90 min incubation with HRP-conjugated secondary antibodies. Protein bands were detected *via* enhanced chemiluminescence (Fude) and imaged using a ChemiDoc XRS + system (Bio-Rad Laboratories). Quantification of band intensity was performed using ImageJ software (NIH). Detailed information on all antibodies used in this study, including catalog numbers and working dilutions, is provided in Table S1.

### NF-κB pathway agonist treatment

To assess whether the antitumor activity of ILL is mediated through NF-κB signaling, a pharmacological rescue assay was performed using the NF-κB activator Diprovocim (MCE). HCCC9810 and RBE cells were pre-exposed to Diprovocim (5 nM, 2 h) and subsequently co-incubated with ILL (20 μm) for 24 h. Additionally, an independent NF-κB activator, PapRIVto (MCE), was applied at 10 μm for 2 h prior to ILL treatment validate the specificity of the rescue effect. Proliferative capacity and protein expression were then examined by EdU incorporation, colony formation, and Western blotting as previously outlined.

### Molecular docking analysis

To explore the potential interaction between ILL and BCL10, molecular docking simulations were performed. The ILL structure was obtained from PubChem (CID: 5318587), and the human BCL10 crystal structure was downloaded from the RCSB Protein Data Bank. Prior to docking, the protein and ligand structures were prepared by removing water molecules and adding polar hydrogens using AutoDockTools v1.5.7 (Scripps Research Institute). Docking was carried out within a grid centered on the predicted active site, and the conformation with the most favorable binding energy was chosen for further analysis. The resulting interactions between ILL and BCL10 were rendered and inspected using PyMOL (Schrödinger Co.).

### Cellular thermal shift assay

To verify the direct interaction of ILL with BCL10 in intact cells, a CETSA was conducted. HCCC9810 and RBE cells were treated with 20 μm ILL or DMSO for 4 h, collected, and resuspended in PBS supplemented with protease inhibitors. To assess protein stability, cell suspensions were divided into aliquots and incubated across a temperature range of 37 to 62 °C for 3 min, followed by cooling for 3 min. Samples then underwent three freeze–thaw cycles to lyse cells. Insoluble material was removed by high-speed centrifugation (20,000*g*, 20 min, 4 °C), and the soluble BCL10 content was evaluated *via* Western blot analysis.

### Plasmid construction and transfection

For BCL10 silencing, shRNAs targeting BCL10 and a scrambled control were cloned into pLKO.1 vectors (Genechem) to generate stable knockdown cell lines (shBCL10) *via* puromycin selection (2 μg/ml). A mutant construct of BCL10 (BCL10-mut) was generated by site-directed mutagenesis, introducing point mutations at Thr-91 and Gln-92, and subsequently subcloned into pcDNA3.1. The BCL10-mut construct was introduced into stable BCL10 knockdown cells (shBCL10) to generate a rescue model, and shRNA-mediated knockdown was constitutive rather than transient. For functional rescue experiments, shBCL10 cells were transfected with plasmids encoding BCL10-mut using Lipofectamine 3000 according to the manufacturer’s instructions. Successful modulation of BCL10 expression was confirmed by Western blot analysis.

### Co-immunoprecipitation

For co-IP, cell lysates were prepared in a detergent-containing IP buffer (20 mM Tris–HCl, pH 7.5; 150 mM NaCl; 1% Triton X-100; 1 mM EDTA) supplemented with protease inhibitors. Following clarification by centrifugation at 14,000*g* for 15 min at 4 °C, an aliquot was set aside as the input control. The supernatant was precleared with Protein A/G magnetic beads for 1 h, then incubated overnight with antibodies specific to BCL10 or control IgG. Immune complexes were captured with fresh beads, washed thoroughly, and released into 2 × SDS loading buffer for downstream Western blot analysis.

### Ubiquitination assay

Cells transfected with WT (BCL10-WT) or mutant (BCL10-Mut) constructs were treated with ILL (20 μm) or DMSO for 12 h. MG132 (20 μm, MCE) was added during the last 6 h to block proteasomal degradation. Lysates were prepared in IP buffer supplemented with 10 mM N-ethylmaleimide to inhibit deubiquitinases. BCL10 was immunoprecipitated as described above, and ubiquitination was detected by Western blotting with an anti-ubiquitin antibody. For linkage-specific ubiquitination analysis, K48-only and K63-only ubiquitin constructs were used to distinguish different ubiquitin chain types.

### Xenograft nude mice model studies

All procedures involving animals were conducted under the approval of the Institutional Animal Care and Use Committee of Zhejiang Chinese Medical University (Approval No. SYXK [Zhejiang] 2018-0012). Male BALB/c nude mice (4–6 weeks old; Shanghai SLAC Laboratory Animal Co) were housed under SPF conditions. Mice were subcutaneously injected in the flank with 2 × 10^6^ HuCCT1 cells in 100 μl PBS. Once tumors reached a uniform size, animals were randomly assigned to receive either vehicle, 1 mg/kg ILL, or 2 mg/kg ILL. Treatments were administered *via* intraperitoneal injection every 3 days for 18 days. Tumor growth and body weights were monitored at 3-days intervals. Tumor length and width were measured using a digital caliper, with length defined as the longest diameter and width as the perpendicular shorter diameter. Tumor volumes were then calculated using the formula: Volume = (length × width^2^)/2. At the study endpoint, mice were euthanized, tumors excised and weighed, and tissues were processed for both histological examination and molecular analyses.

### Histology and immunohistochemistry

Tumor tissues were harvested, fixed in 4% paraformaldehyde, dehydrated through ascending ethanol concentrations, and embedded in paraffin for sectioning. Sections were prepared for either H&E staining or immunohistochemical detection. For immunohistochemistry, deparaffinized sections underwent antigen retrieval in citrate buffer (pH 6.0), endogenous peroxidase was blocked with 3% hydrogen peroxide, and nonspecific sites were blocked with 5% goat serum. Primary antibodies against Ki67 or PCNA were applied overnight at 4 °C, followed by HRP-conjugated secondary antibody incubation. Signal development was carried out using DAB, counterstaining with hematoxylin was performed, and images were captured using a Nikon light microscope.

### Statistical analysis

All numerical data were subjected to statistical evaluation using GraphPad Prism 9.0 (GraphPad Software) and are presented as mean ± SD. Two-group comparisons utilized a two-tailed Student’s *t* test, while analyses involving three or more groups were conducted *via* one-way ANOVA followed by Bonferroni *post hoc* test to account for multiple comparisons. A threshold of *p* < 0.05 was used to determine statistical significance.

## Data availability

The RNA-seq data generated in this study have been deposited in the Gene Expression Omnibus (GEO) under accession number GSE318264. Other data that support the findings of this study are available from the corresponding author upon reasonable request.

## Supporting information

This article contains supporting information.

## Conflict of interest

The authors declare that there are no conflicts of interest with the contents of this article.
