# Supplementary material for: Isolinderalactone suppresses the progression of cholangiocarcinoma by modulating the CARMA1-BCL10-MALT1 signalosome
Source: J Biol Chem. 2026 May 15;302(7):113162. doi: 10.1016/j.jbc.2026.113162 (PMC13273830; doi:10.1016/j.jbc.2026.113162)
Supplement: Supplementary Material [file mmc1.docx]

**Isolinderalactone Suppresses the Progression of Cholangiocarcinoma by Modulating the CARMA1-BCL10-MALT1 Signalosome**

Wangyang Chen^1,2,3,4†^, Dongchao Xu^1,2,3,4†^, Qiang Liu^1,2,3,4†^, Xuehui Wang^1,2,3,4^, Chenshan Xu^1,2,3,4^, Yuanling Zhu^1,2,3,4^, Wenjun Lin^1,2,3,4^, Hongchen Zhang^1,2,3,4^, Xiaofeng Zhang^1,2,3,4*^, Jianfeng Yang^1,2,3,4**^, Hongzhang Shen^1,2,3,4***^

^1^Department of Gastroenterology, Affiliated Hangzhou First People's Hospital, Westlake University School of Medicine, Hangzhou, Zhejiang Province, 310000, China

^2^Hangzhou Institute of Digestive Diseases, Hangzhou, Zhejiang Province, 310003, China

^3^Key Laboratory of Integrated Traditional Chinese and Western Medicine for Biliary and Pancreatic Diseases of Zhejiang Province, Hangzhou, Zhejiang Province, 310003, China

^4^Key Laboratory of Clinical Cancer Pharmacology and Toxicology Research of Zhejiang Province, Hangzhou, Zhejiang, 310003, China

*****Corresponding author at:** Affiliated Hangzhou First People’s Hospital, Westlake University School of Medicine, 261 Huansha Road, Hangzhou, Zhejiang, 310006, China. Email address: shenhongzhang@zju.edu.cn. Tel/Fax: 86-0571-87914773.

****Corresponding author at:** Affiliated Hangzhou First People’s Hospital, Westlake University School of Medicine, 261 Huansha Road, Hangzhou, Zhejiang, 310006, China. Email address: yangjianfeng@hospital.westlake.edu.cn. Tel: 86-13454132186.

***Corresponding author at:** Affiliated Hangzhou First People’s Hospital, Westlake University School of Medicine, 261 Huansha Road, Hangzhou, Zhejiang, 310006, China. Email address: [zxf837@tom.com](mailto:zxf837@tom.com). Tel: 86-0571-56005600.

† Wangyang Chen, Dongchao Xu, and Qiang Liu contributed equally.

**Supplementary Tables**

| Antibody | Catalog number &Vendor | Uses |
| --- | --- | --- |
| Ki67 | ET1609-34 (HUABIO) | Western blot (1:1000) Immunohistochemistry (1:500) |
| PCNA | 60097-1-Ig (Proteintech) | Western blot (1:5000) Immunohistochemistry (1:500) |
| β-Actin | ab8227 (Abcam) | Western blot (1:1000) |
| N-Cadherin | ab76011 (Abcam) | Western blot (1:5000) |
| E-Cadherin | ab40772 (Abcam) | Western blot (1:1000) |
| Vimentin | ET1610-39 (HUABIO) | Western blot (1:20000) |
| GAPDH | 2118 (Cell Signaling Technology) | Western blot (1:1000) |
| Caspase 3 | ab32351 (Abcam) | Western blot (1:5000) |
| Bcl 2 | ab182858 (Abcam) | Western blot (1:2000) |
| Cyclin A2 | 27242-1-AP (Proteintech) | Western blot (1:1000) |
| Cyclin B1 | ab181593 (Abcam) | Western blot (1:2000) |
| Cyclin D1 | ab40754 (Abcam) | Western blot (1:1000) |
| Cyclin E1 | ET1612-16 (HUABIO) | Western blot (1:1000) |
| IκBα | ab32518 (Abcam) | Western blot (1:1000) |
| p-IκBα (Ser32) | ab92700 (Abcam) | Western blot (1:1000) |
| p65 | ab32536 (Abcam) | Western blot (1:1000) |
| p-p65 (Ser468) | ab264271 (Abcam) | Western blot (1:2000) |
| CARMA 1 | ab124730 (Abcam) | Western blot (1:1000) |
| BCL 10 | ab33905 (Abcam) | Western blot (1:1000) |
| MALT1 | ab33921 (Abcam) | Western blot (1:1000) |
| Ubiquitin | ab7780 (Abcam) | Western blot (1:1000) |
| Ubiquitin (linkage-specific K63) | ab179434 (Abcam) | Western blot (1:1000) |
| Ubiquitin (linkage-specific K48) | ab140601 (Abcam) | Western blot (1:1000) |

**Table S1. Antibodies used in the study.**

**Table S2. Primer sequences used in this study for quantitative real-time PCR analysis**

| Gene | Sequences (5’-3’) |
| --- | --- |
| β-actin | Forward: ACAGAGCCTCGCCTTTGC Reverse: GATATCATCATCCATGGTGAGCTGG |
| BCL2 | Forward: GATAACGGAGGCTGGGATGC Reverse: TCACTTGTGGCCCAGATAGG |
| GADD45B | Forward: CCAGTCCTTCTGCTGTGACAA Reverse: TCCGTGTGAGGGTTCGTGA |
| TRAF1 | Forward: CTTGAGGTCACCCAGACACTC Reverse: GATGGTGACTGAAGGCTTCCTG |

Supplementary Figures


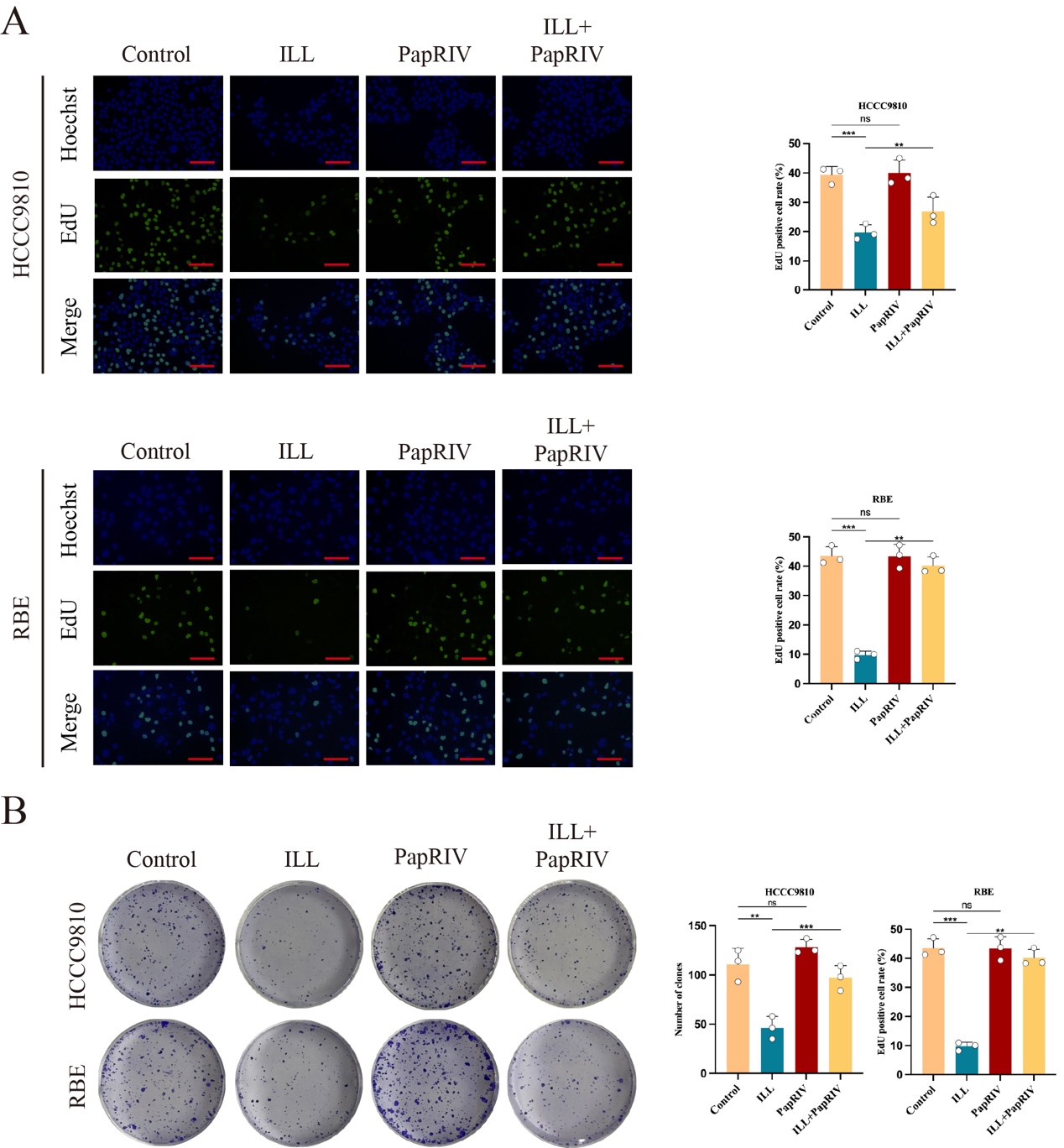


**Figure S1.** PapRIV counteracts the inhibitory effects of ILL on CCA cell proliferation. (A) EdU staining and positive rate analysis in CCA cells treated with ILL, PapRIV, or the combination of ILL and PapRIV (mean ± SD; one-way ANOVA followed by Bonferroni post hoc test; n = 3 biologically independent experiments). Scale bars = 200 μm. (B) Colony formation assays and quantification of colony numbers in CCA cells treated with ILL, PapRIV, or the combination of ILL and PapRIV (mean ± SD; one-way ANOVA followed by Bonferroni post hoc test; n = 3 biologically independent experiments). **p* < 0.05, ***p* < 0.01, ****p* < 0.001.


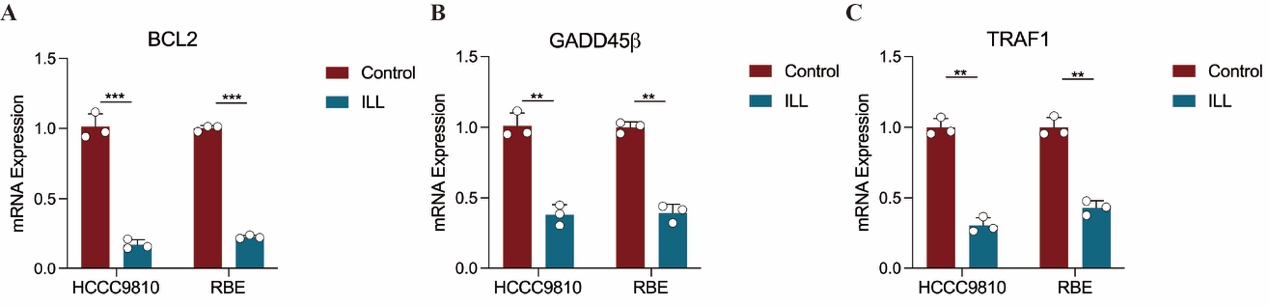


**Figure S2.** ILL suppresses NF-κB–dependent transcriptional activity. Relative mRNA expression levels of NF-κB target genes BCL2 (A), GADD45β (B), and TRAF1 (C) were measured by quantitative PCR in HCCC9810 and RBE cells following ILL treatment (mean ± SD; one-way ANOVA followed by Bonferroni post hoc test; n = 3 biologically independent experiments). **p* < 0.05, ***p* < 0.01, ****p* < 0.001.


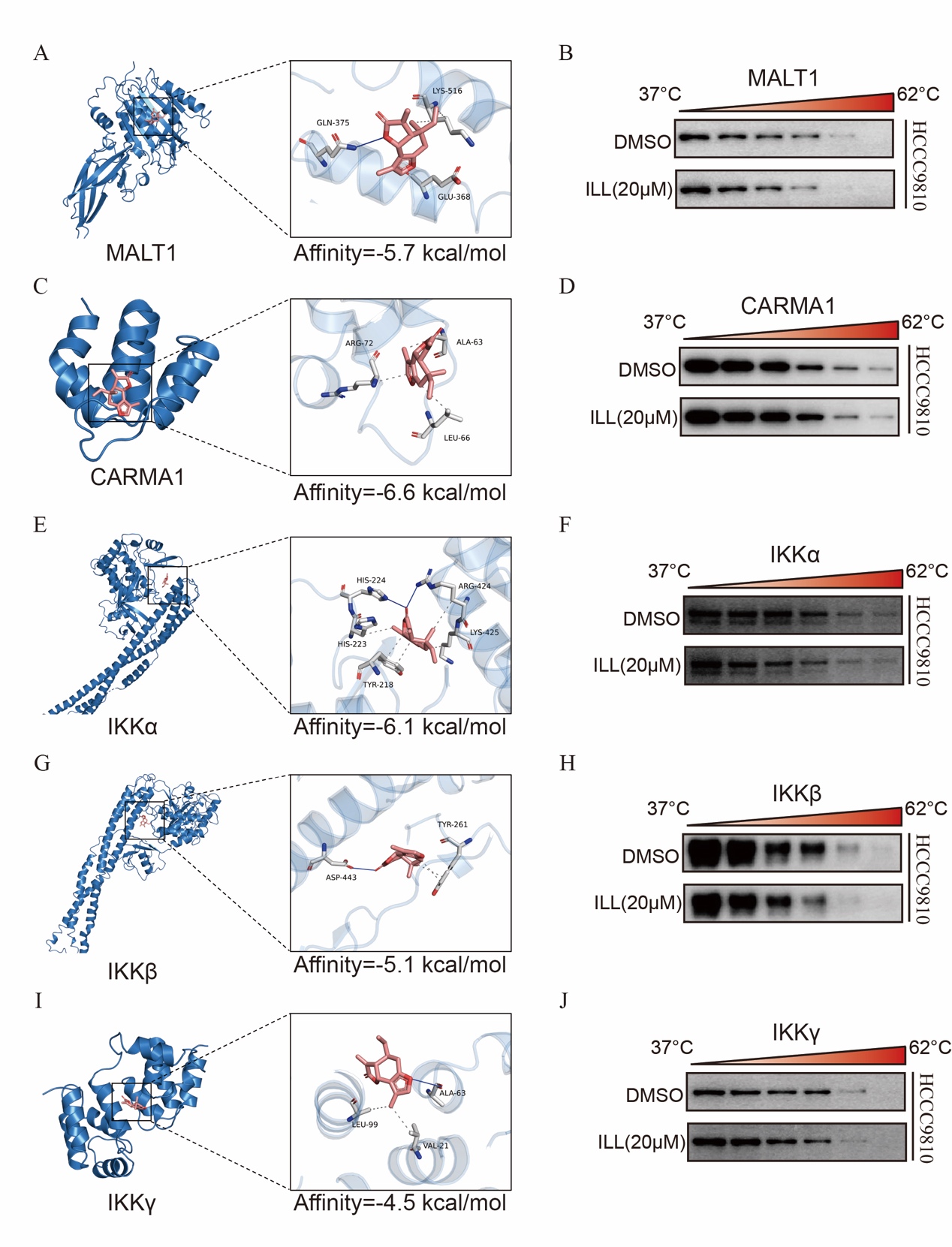


**Figure S3.** Evaluation of ILL binding and target engagement with other CBM and IKK components. Molecular docking models, calculated binding affinities, and CETSA results of ILL with MALT1 (A-B), CARMA1 (C-D), IKKα (E-F), IKKβ (G-H), and IKKγ (I-J).


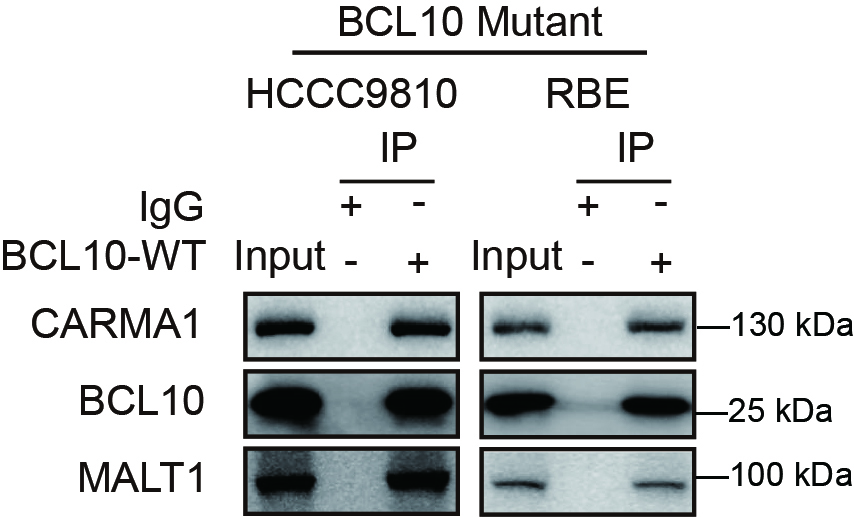


**Figure S4.** BCL10 mutation does not affect basal CBM complex assembly. Co-immunoprecipitation assays were performed in HCCC9810 and RBE cells expressing BCL10-mut without ILL treatment. BCL10 was immunoprecipitated, and the interaction with CARMA1 and MALT1 was analyzed by immunoblotting. BCL10-mut retained its interaction with CARMA1 and MALT1, comparable to wild-type BCL10. IgG served as a negative control.


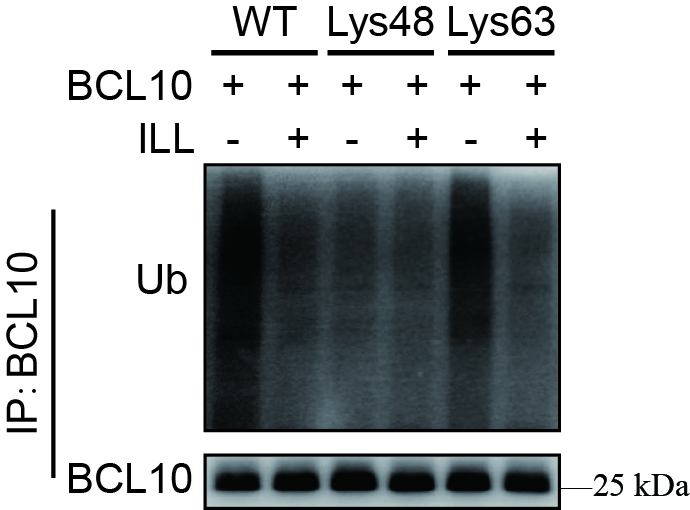


**Figure S5.** ILL selectively inhibits K63-linked ubiquitination of BCL10. HCCC9810 cell was transfected with BCL10 together with wild-type (WT), K48-only, or K63-only ubiquitin constructs and treated with or without ILL. BCL10 was immunoprecipitated, and ubiquitination was detected by immunoblotting with an anti-ubiquitin antibody. The lower panel shows immunoblotting of BCL10 as a loading control for IP efficiency. ILL markedly reduced WT and K63-linked ubiquitination of BCL10, while having minimal effect on K48-linked ubiquitination.
